# Involvement of TORC2, a CREB co-activator, in the *in vivo*-specific transcriptional control of HTLV-1

**DOI:** 10.1186/1742-4690-6-73

**Published:** 2009-08-11

**Authors:** Shiwen Jiang, Takefumi Inada, Masakazu Tanaka, Rika A Furuta, Koh Shingu, Jun-ichi Fujisawa

**Affiliations:** 1 Department of Microbiology Kansai Medical University, Moriguchi, Osaka 570-8506, Japan; 2 Department of Anesthesiology, Kansai Medical University, Moriguchi, Osaka 570-8506, Japan; 3 Japanese Red Cross Osaka Blood Center, Morinomiya, Joto-ku, Osaka 536-8505, Japan

## Abstract

**Background:**

Human T-cell leukemia virus type 1 (HTLV-1) causes adult T -cell leukemia (ATL) but the expression of HTLV-1 is strongly suppressed in the peripheral blood of infected people. However, such suppression, which may explain the long latency in the development of ATL, is readily reversible, and viral expression resumes quickly with *ex vivo *culture of infected T -cells. To investigate the mechanism of *in vivo -*specific transcriptional suppression, we established a mouse model in which mice were intraperitoneally administered syngeneic EL4 T -lymphoma cells transduced with a recombinant retrovirus expressing a GFP-Tax fusion protein, Gax, under the control of the HTLV-1 enhancer (EL4-Gax).

**Results:**

*Gax *gene transcription was silenced *in vivo *but quickly up-regulated in *ex vivo *culture. Analysis of integrated *Gax *reporter gene demonstrated that neither CpG methylation of the promoter DNA nor histone modification was associated with the reversible suppression. ChIP-analysis of LTR under suppression revealed reduced promoter binding of TFIIB and Pol-II, but no change in the binding of CREB or CBP/p300 to the viral enhancer sequence. However, the expression of TORC2, a co-activator of CREB, decreased substantially in the EL4-Gax cells *in vivo*, and this returned to normal levels in *ex vivo *culture. The reduced expression of TORC2 was associated with translocation from the nucleus to the cytoplasm. A knock-down experiment with siRNA confirmed that TORC2 was the major functional protein of the three TORC-family proteins (TORC1, 2, 3) in EL4-Gax cells.

**Conclusion:**

These results suggest that the TORC2 may play an important role in the *in vivo *-specific transcriptional control of HTLV-1. This study provides a new model for the reversible mechanism that suppresses HTLV-1 expression *in vivo *without the DNA methylation or hypoacetylated histones that is observed in the primary cells of most HTLV-1 -infected carriers and a substantial number of ATL cases.

## Background

Human T-cell leukemia virus type 1 (HTLV-1), a life-long persistent CD4+T -lymphotropic retrovirus, causes an aggressive mature T -cell malignancy termed "adult T-cell leukaemia" (ATL) [1,2] and an inflammatory disease of the central nervous system known as HTLV-1-associated myelopathy/tropical spastic paraparesis (HAM/TSP) [3,4]. HTLV-1 infects 10–20 million people worldwide; 2–3% of infected individuals develop ATL, and a further 0.25–3% develop HAM/TSP.

Tax protein, encoded by the HTLV-1 pX region [5], is closely associated with the development of these diseases by triggering in a pleiotropic manner viral transcription [6-9] and by deregulating the expression of cellular genes [10,11]. However, the expression of viral genes, including Tax, is almost completely suppressed in the peripheral blood of infected people [12]. This may explain the long latency in the development of ATL and other HTLV-1 related diseases. It has been assumed that there is a specific mechanism for this *in vivo *-specific suppression, because gene expression of HTLV-1 in peripheral blood cells from infected people, with the exception of two-thirds of ATL patients [13], resumes quickly when the infected cells are moved to *in vitro *conditions, without any stimulation [12]. Such reversible control of the gene expression should benefit HTLV-1 because Tax protein harbors several strong epitopes for cytotoxic T -cells [14]. Thus, the transient expression of Tax is essential for the propagation of viral infection and/or infected cells under strict surveillance by the host immune system [15], the efficiency of which may vary among individuals [16]. In contrast, evading the suppressed state leading to the reactivation of viral gene expression may be a key step in the development of HTLV-1 associated diseases.

DNA methylation accumulated in HTLV-1 5'-LTR silences viral gene transcription in leukemic cells [13,17]. However, further analysis revealed that viral gene transcription is silenced in most carriers, and in about 20% of ATL cases, despite no or only partial methylation of the 5'-LTR [18]. Furthermore, in the case of ATL, transcriptional silencing was observed regardless of the acetylation of histones H3 and H4, markers of active transcription, in the 5'-LTR [18]. Thus, a reversible mechanism that suppresses viral gene transcription without DNA methylation or hypoacetylated histones in 5'-LTR has been postulated but remains to be clarified.

As observed in other retroviruses, transcription of HTLV-1 is under the control of an enhancer/promoter located in its LTR. The U3 region in the HTLV-1 LTR harbors an enhancer element consisting of three 21 -bp direct repeats that are activated exclusively in the presence of Tax. In the center of each 21 -bp enhancer sequence there are Tax-responsive elements (TRE) or viral cyclic AMP response elements (CRE) [9,19], to which a variety of enhancer binding proteins, including members of the CREB/ATF family, bind, with or without Tax protein [20]. Among them, CREB has been implicated as the primary player in both basal and Tax-activated HTLV-1 transcription [21,22]. CREB stimulates HTLV-1 viral transcription by binding to the viral CRE and interacts with Tax, which is also associated with the GC-rich sequences immediately flanking the viral CRE, and recruits CBP/p300 to form a Tax/CREB/CBP/p300/DNA quaternary complex [23,24].

In contrast, proteins belonging to another recently identified family of CREB cofactors, termed "transducers of regulated CREB activity" (TORCs) [25,26] have been suggested to enhance HTLV-1 transcription, alone or in combination with Tax, in a CREB-dependent manner *in vitro *[27,28]. TORCs were originally found in the CREB -dependent, but pCREB(phosphor-CREB)-independent, activation of cellular genes [26]. The recruitment of TORCs to the promoter does not appear to modulate CREB DNA binding activity, but rather enhances the interaction of CREB with the TAFII130 component of TFIID [26]. Among three members of the TORC-family protein, the activity of TORC2 is tightly regulated by phosphorylation at Ser 171, which promotes the export of the protein into the cytoplasm and its degradation [29].

To gain insights into the mechanism of this *in vivo *-specific transcriptional suppression, we established a mouse model in which mice were intraperitoneally administered syngeneic EL4 T -lymphoma cells transduced with a recombinant retrovirus expressing GFP-Tax fusion protein under the control of the HTLV-1 enhancer (EL4-Gax). Gax protein retains the properties of Tax as a transcriptional transactivator and also as an antigen, providing epitopes for CTL [30]. Furthermore, *Gax *expression in EL4-Gax cells is suppressed *in vivo *but is quickly up-regulated in *ex vivo *culture, thus modeling the activity of HTLV-1 -infected cells in asymptomatic carriers [30]. The present study analyzed epigenetic modifications and factors in the integrated HTLV-1 promoter/enhancer in EL4-Gax cells *in vivo *as well as *ex vivo*. We found that reduced expression of TORC2, but not of CREB or its phosphorylated form (pCREB), was responsible for the suppression of viral gene expression *in vivo*.

## Results

### *Gax *expression *in vivo *was suppressed at the level of transcription

**Figure 1:**
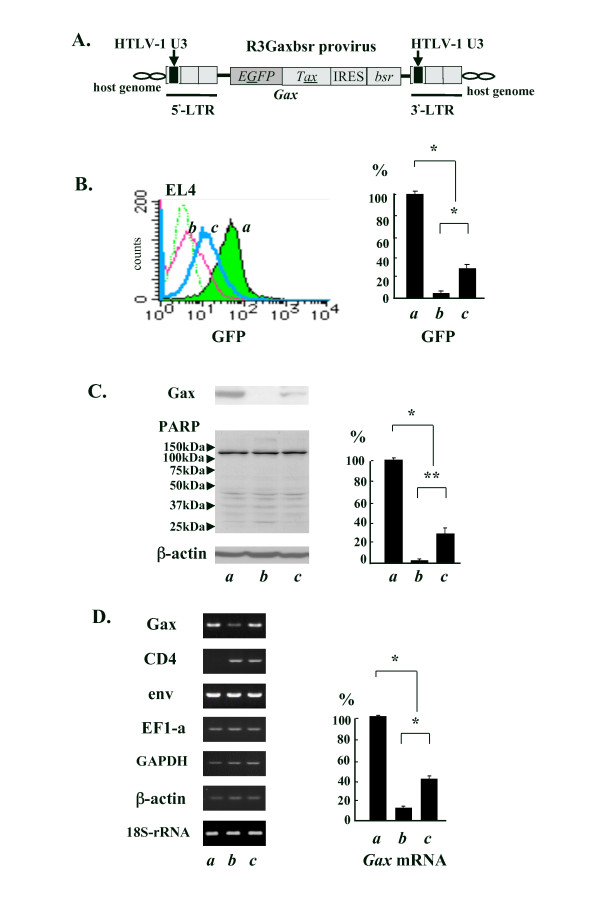
**A mouse model system with EL4-Gax cells**. A. The structural organization of the R3Gaxbsr genome in EL4-Gax cells [29]. The EGFP coding sequence was fused with *tax *cDNA at the 5'-end, resulting in *Gax*. The *Gax *gene was linked with a drug resistance gene, bsr, by an internal ribosome entry site (IRES). The U3 region in the MLV LTR was replaced with that in the HTLV-1 LTR. 1 × 10^6 ^of EL4-Gax cells cultured *in vitro *(*in vitro*, *a*) were injected into peritoneal cavity of a syngenic C57BL/6J mouse. 3 weeks after challenge, cells were collected from ascitic fluids (*in vivo, b*) and transferred to the *in vitro *culture condition for 48 hours (*ex vivo, c*). B. Left, the expression of Gax protein in living cells was monitored as the intensity of GFP fluorescence by fluorescent-activated cell sorter (FACS); Right, statistical analysis of the GFP mean fluorescent intensity (GFP mfi), after deducting EL4 cells background level. EL4, parent cell line. C. Left, cell lysates were subjected to Western blot analysis with anti-Tax serum, PARP, an indicator of apoptosis or necrosis exhibiting the signature 89 kDa or 50 kDa fragment respectively to see if the *in vivo *cells were healthy or not, or anti-β-actin antibody as loading control; Right, quantification of Gax and normalized to β-actin with a densitometry software program (NIH-image). D. Left, RT-PCR analysis of several viral and cellular mRNAs.; Right, Real-time PCR analysis of *Gax *mRNA expression and normalized to 18S ribosomal RNA in EL4-Gax cells with SYBR Green. Error bars indicate SEMs. Data were obtained from four independent experiments analyzing one mouse per experiment, and statistical analysis of the data was performed between the *in vivo *or *ex vivo *against the *in vitro*. *; p < 0.01. **; p < 0.05.

EL4-Gax cell was established by transducing with an MLV-based retrovirus vector expressing the GFP-fused Tax (*Gax*), in which the U3 region of the 3' LTR was replaced by that of HTLV-1 to ensure the Tax-dependent transcriptional control of HTLV-1 (Fig. 1A), and the characteristics of Gax protein as a transactivator were shown to be retained as previously reported [30]. Expression of *Gax *gene under the control of HTLV-1 LTR in EL4-Gax cells grown in the peritoneal cavity of mice and cultured *in vitro *was directly monitored by the intensity of GFP fluorescence using a fluorescent-activated cell sorter (FACS). This demonstrated the *in vivo *-specific suppression of Tax expression (Fig. 1B) [30].

Immunoblot analysis confirmed that *in vivo *protein expression of *Gax *was abolished in cells, and the expression was reactivated in *ex vivo *culture (Fig. 1C, Gax). The reduction of Gax protein is not simply due to a severe growth condition inducing cell death since the proteolytic cleavage of poly(ADP-ribose) polymerase, which is known to be a sensitive marker of apoptosis [31] and necrosis [32], was not observed (Fig. 1C, PARP).

The expression of *Gax *mRNA was analyzed using quantitative reverse transcription polymerase chain reaction (RT-PCR) to determine whether the suppression of Tax expression was controlled at the level of transcription (Fig. 1D). The transcriptional suppression *in vivo *is specific for the Gax gene because no suppression was observed in the expression of cellular genes such as EF1-a, GAPDH, β-actin, 18S-ribosomal RNA and endogenous retrovirus. On the contrary, gene expression of CD4 was upregulated *in vivo*, while it was silenced in EL4-Gax cells grown *in vitro*. Real-time PCR analysis of *Gax *cDNA prepared from total RNA in EL4-Gax cells demonstrated that the expression of *Gax *mRNA was reduced *in vivo *and recovered after *ex vivo *culturing to a level comparable with that before peritoneal inoculation of the cells. Thus, Gax expression *in vivo *was suppressed transcriptionally.

### CpG methylation is not associated with the suppression of the *Gax *gene

**Figure 2:**
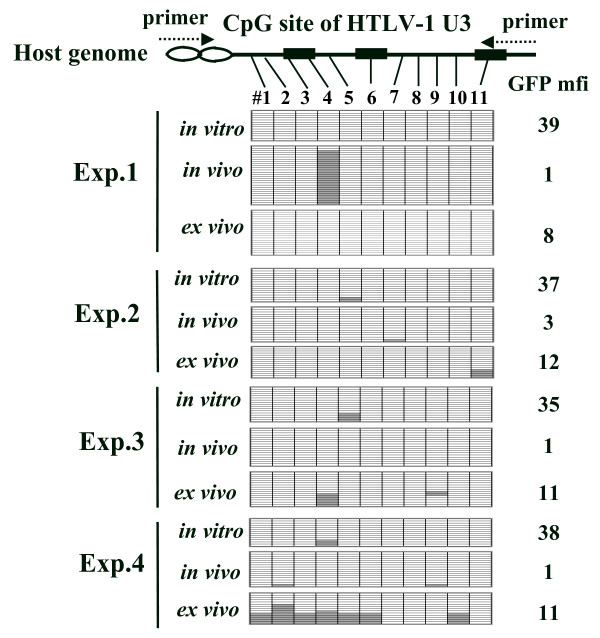
**CpG methylation of the enhancer/promoter region of provirus DNA in EL4-Gax cells**. Top: locations of CpG sites (#1–11) in the HTLV-1 U3 region studied in this experiment. The sense primer is complementary to the mouse genomic sequence flanking the 5'-LTR of provirus at the integration site, and the anti-sense primer is complementary to the junction sequence between the HTLV-1 and MLV U3 regions. The three 21 -bp enhancer sequences are indicated as boxes. Bottom: results of bisulfite genomic sequencing analysis of four independent experiments. Methylated and unmethylated CpG sites are expressed as filled and open rectangles, respectively. Amplified PCR products were subcloned into pGEM-T vector, and the nucleotide sequences of at least 13 clones were determined. GFP mfi: the GFP mean fluorescent intensity of EL4-Gax cells used for bisulfite genomic sequencing analysis.

Because complete- or hyper- methylation of cytosine residues at the CpG sites in the promoter region of the HTLV-1 5'-LTR is associate with transcriptional suppression in infected cell lines, the level of CpG methylation in the LTR U3 region at 5' site of *Gax*-reporter genome was examined in EL4-Gax cells. There are 11 possible CpG methylation sites in the U3 region of HTLV-1, but only low levels of methylation were observed in four independent experiments. Although one case (experiment 1 in Fig. 2) showed heavy methylation at a single CpG site in EL4-Gax cells *in vivo*, little or no methylation was detected at this site in the other experiments. In the other three experiments, less methylation was observed in EL4-Gax cells *in vivo *(where *Gax *expression was suppressed) than in cells grown *in vitro *or *ex vivo*. Thus, no CpG methylation specific and consistent with that in the *in vivo *cells was detected (Fig. 2). These results indicate that the suppression of *Gax *gene expression *in vivo *is not explained by CpG methylation in the enhancer sequence, suggesting the involvement of other mechanism(s). This is consistent with a previous analysis in which no or partial methylation was associated with silencing in the peripheral blood cells of HTLV-1 carriers, as well as in significant number of ATL cases, whereas transcriptional suppression of HTLV-1 in ATL cell lines and some ATL leukemic cells was explained by hypermethylation of the 5'-LTR [18].

### Binding of CREB and pCREB to the HTLV-1 enhancer

CREB has been implicated as the primary player in both basal and Tax-activated HTLV-1 transcription [24]. CRE-dependent transcription is generally explained by the recruitment of histone acetylating proteins, CBP/p300, to the enhancer region of genes through an interaction with CREB protein, which binds to the CRE sequence, and acetylation of histones, opening the chromatin and providing access to basic transcriptional factors including RNA polymerase. Thus, since the reduction of recruitment of either factor to the promoter region might result in the suppression of transcription, a chromatin immunoprecipitation (ChIP) assay was used to analyze the binding of these factors to the U3 region of the 5'-LTR.

**Figure 3:**
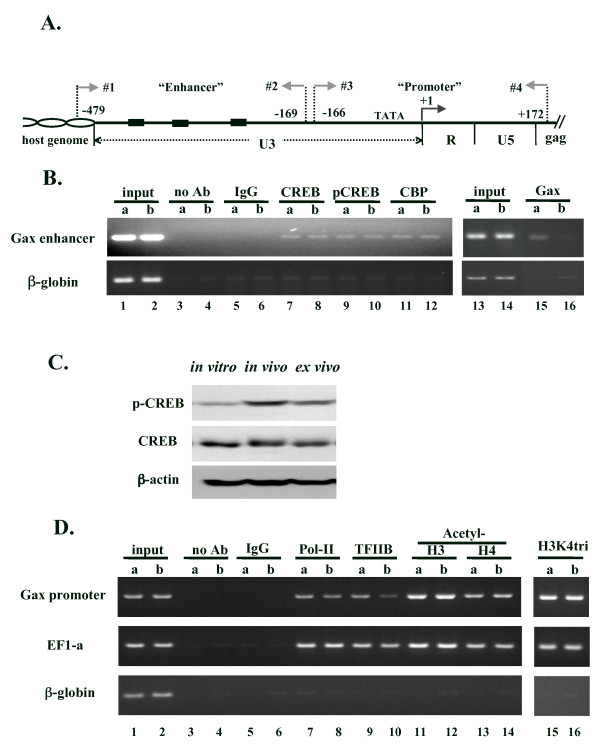
**ChIP analysis of the enhancer/promoter region in *Gax *provirus**. A. Schematic representation of the 5'-LTR in the R3Gaxbsr reporter gene. The three 21 -bp enhancer sequences (boxes), the TATA sequence, and the transcription start site (+1) are shown. Primers for PCR are indicated by arrows. The 5'- and 3'-ends of amplified DNA are denoted as the nucleotide positions relative to the transcription start site. Primers #1 and #2 amplify the enhancer region, and primers #3 and #4 amplify the promoter region of *Gax*-5'-LTR. B. Binding of CREB, phosphor-CREB and CBP to the *Gax *enhancer region was constant in EL4-Gax cells *in vitro *(a) and *in vivo *(b)(left), but the enhancer binding of Gax was reduced when EL4-Gax cells were grown *in vivo *(right). C. Expression of CREB protein in EL4-Gax cells. Anti-CREB1, anti-phosphor-CREB antibodies were used to detect proteins in EL4-Gax cells grown *in vitro*, *in vivo*, and *ex vivo*. Equivalent protein loading was confirmed by stripping and re-probing the blot with an anti-β-actin antibody. D. Binding of acetylated histone 3 at Lys-9, 14 (H3), acetylated histone 4 at Lys- 5, 8, 12, 16 (H4) and trimethylated histone 3 at Lys-4 (H3K4tri) to the *Gax *enhancer region was not changed in EL4-Gax cells either *in vitro *(a) or *in vivo *(b), but the promoter binding of the basic transcription factor TFIIB and of RNA polymerase II (Pol-II) was reduced when EL4-Gax cells were grown *in vivo*. Factors binding to promoters of EF-1a and β-globin are presented as positive and negative controls, respectively.

Enhancer binding of CREB and pCREB was first examined in EL4-Gax cells either *in vivo *(b) or under *in vitro *(a) culture conditions. As shown in Figure 3B (lanes 7–10), no significant difference was observed in the amount of CREB or pCREB in complex with the enhancer DNA at the 5'-LTR of the provirus. CBP functions as a cofactor by being tethered to DNA through either pCREB or CREB, in association with Tax, to acetylate histone proteins. Binding of CBP to the HTLV-1 enhancer was observed but showed a similar intensity of protein binding (Fig. 3B, lanes 11, 12).

As Gax is expressed in EL4-Gax cells *in vitro*, it was of interest whether Gax is associated with the enhancer DNA. ChIP assay was performed with antibody against GFP, which recognizes the Gax protein. Consistent with the protein expression, Gax was associated with the enhancer DNA in EL4-Gax cells grown *in vitro *(lane 15) but not in *in vivo *cells (lane 16), where the expression of Gax protein was decreased. Tax recruits CBP to the HTLV-1 enhancer by tethering with CREB at the CRE sequence; however, the enhancer binding of CBP remained unchanged in the absence of Tax (Fig 3B, lane 11, 12). In this respect, it is noteworthy that phosphorylation of CREB protein, which leads to a complex formation between CREB and CBP, is increased in EL4-Gax cells grown *in vivo *(Fig 3C). Thus, pCREB seems to be involved in the sustained enhancer binding of CBP in the absence of Tax.

Although the amount of pCREB was increased in EL4-Gax cells grown *in vivo*, no significant difference was observed in the amount of pCREB binding to the enhancer DNA in cells either *in vivo *or under *in vitro *culture conditions. Since pCREB has been demonstrated to preferentially bind to the enhancer sequence of HTLV-1 in a complex with Tax [33], Tax might have selectively incorporated pCREB in the complex.

### Modifications of histones H3 and H4

Activated transcription is associated with histone acetylation in the chromatin of the respective genes; thus, histone acetylation at the promoter region of the provirus was analyzed using a ChIP assay, with antibodies against acetylated histones H3 and H4. Unexpectedly, this analysis revealed that histones at the LTR of the HTLV-1 provirus were equally acetylated in EL4-Gax cells (Fig. 3D, lanes 11–14), either *in vivo *(b) and *in vitro *(a), whereas RNA expression from the HTLV-1 promoter in these cells differed substantially (Fig. 1). Methylation of histone H3 at the lysine residue was also analyzed, because this methylation is closely linked with transcriptional activation. However, no change was observed in the methylation of histone H3 in the promoter region of the provirus (Fig. 3D, lanes 15–16). Thus, the *in vivo *-specific transcriptional repression of the HTLV-1 promoter was not associated with an altered level of chromatin modification. These results are consistent with the previous finding that gene silencing of HTLV-1 in an ATL case was observed regardless of hyperacetylation of histones H3 and H4 in the promoter [18].

### Recruitment of basal transcription machinery to the proviral promoter

The recruitment of RNA polymerase II (Pol-II) and TFIIB, a key general transcription factor in forming and stabilizing the early initiation complex [34], was analyzed to determine whether suppression was present in the formation of the transcription initiation complex in the 5'-LTR promoter. Although binding of TFIIB (Fig. 3D, lanes 9, 10) and Pol-II (Fig. 3D, lanes 7,8) to the constitutive promoter of the EF-1a gene as positive controls was observed equally in the ChIP assay, a substantial reduction in the binding of these factors to the provirus promoter sequence was detected under condition of suppressed *Gax *expression in comparison with EL4 -Gax cells in the *in vitro *culture (37 ± 5% for TFIIB and 47 ± 5% for Pol-II). These results suggest that the loss of recruitment of basal transcription factors is associated, at least in part, with the suppression of *Gax *expression *in vivo*, regardless of the constitutive binding of CREB-CBP/p300 to the enhancer DNA.

### Expression of TORC1 and TORC2 is repressed in EL4-Gax cells *in vivo*

In addition to the CREB-CBP/p300 pathway, another family of CREB cofactors, TORCs, has been recently identified as activating CREB-dependent, but pCREB-independent, transcription [25,26], including that of HTLV-1, with or without Tax [27,28]. Thus, we next examined the involvement of TORCs in transcriptional control *in vivo*.

**Figure 4:**
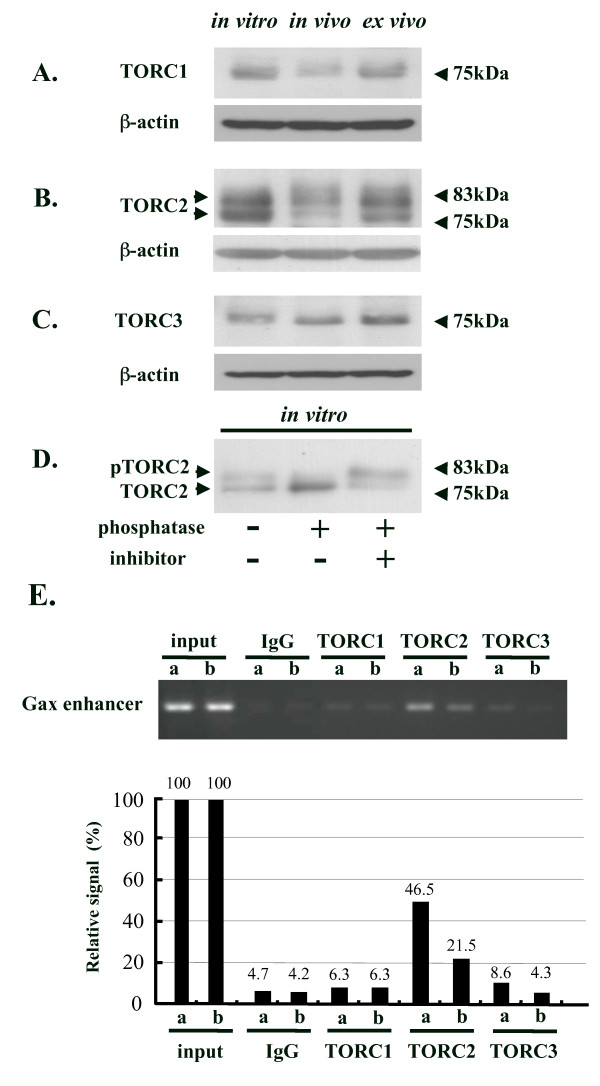
**Expression of TORC proteins in EL4-Gax cells**. Anti-TORC1 (A), anti-TORC2 (B), and anti-TORC3 (C) antibodies were used to detect each protein in EL4-Gax cells grown *in vitro*, *in vivo*, and *ex vivo*. Equivalent protein loading was confirmed by stripping and re-probing the blot with an anti-β-actin antibody. Apparent molecular weights of marker protein are indicated. D. Phosphorylation of TORC2. Protein from EL4-Gax cells was incubated with or without rAPid Alkaline Phosphatase (see methods in detail). E. ChIP analysis of TORCs in EL4-Gax cells *in vitro *(a) and *in vivo *(b). Little or no binding of TORC1 and TORC3 to the *Gax *enhancer region was observed *in vitro *(a) or *in vivo *(b), but the binding of TORC2 to the *Gax *enhancer region was high *in vitro *(a) and reduced when EL4-Gax cells were grown *in vivo *(b).

The TORC family consists of three proteins, TORC1, TORC2, and TORC3; expression of these proteins in EL4-Gax cells was assessed by immunoblot analysis using antibodies against each. All three TORC proteins were detected in the cell lysate prepared from EL4-Gax cells, at molecular weights of 75, 77/82, and 75 kDa respectively. Consistent with previous reports, all TORC proteins appeared to migrate as multiple bands, likely because of they are phosphorylated. In particular, TORC2 protein was composed of two distinct bands, of which the slower migrating band was previously shown to be a phosphorylated form of the faster migrating species. In fact, alkaline phosphatase treatment of cellular lysate from EL4-Gax cells reduced the intensity of the slower migrating band and resulted in the increase of the faster migrating band (Fig. 4D).

When expression of the TORC proteins in EL4-Gax cells grown *in vitro*, *in vivo*, and *ex vivo *was compared, the amounts of TORC1 and TORC2 were reduced significantly under *in vivo *growth conditions, and they recovered to some extent upon their *ex vivo *culturing (Fig. 4A, B). In contrast, the expression of TORC3 increased little, if any, in *in vivo *or *ex vivo *conditions (Fig. 4C). Because a previous report demonstrated that the suppression of TORC1, TORC2 or TORC3 expression by siRNA resulted in reduced transcription from the HTLV-1 LTR [27], it seems likely that reduced expression of TORC1 and/or TORC2 is involved in the suppression of *Gax *gene expression in *in vivo *conditions. It is noteworthy that the reduction of the unphosphorylated TORC2 protein was more significant than that of the phosphorylated form, because the former is an active form of TORC2 retained in the nucleus.

### Binding of TORC proteins to the HTLV-1 enhancer

As TORC proteins are recruited to enhancer DNA in combination with CREB protein to activate CRE-dependent transcription of HTLV-1, a ChIP assay was used to analyze whether these proteins are associated with the U3 region of the 5'-LTR in EL4-Gax cells grown *in vitro *and *in vivo*. As shown in Figure 4E, recruitment of TORC2 and TORC3 proteins to the enhancer sequence was demonstrated in EL4-Gax cells *in vitro *and both of the bindings were substantially reduced in *in vivo *cells, where little or no binding of TORC1 to enhancer DNA was observed. As judged by densitometoric analysis, TORC2 appears to be the main TORC protein that is associated with the enhancer sequence of HTLV-1 in EL4-Gax cells, and the reduced enhancer binding of TORC2 in cells grown *in vivo *was in good agreement with the transcriptional suppression of *Gax in vivo*.

### TORC2 is primarily involved in the transcriptional control of the HTLV-1 promoter in EL4-Gax cells

**Figure 5:**
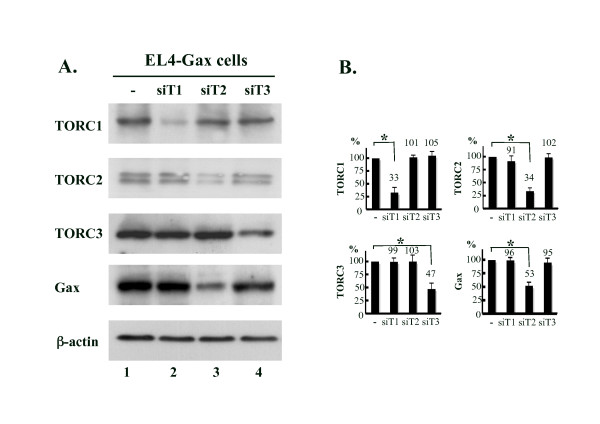
**Effect of the knock-down of TORC genes on the expression of Gax in EL4-Gax cells**. A. Expression of TORC proteins and Gax in EL4-Gax cells transduced with retrovirus (for TORC1 and 3) or lentivirus (for TORC2) vectors encoding siRNA against each TORC gene. Expression of the siRNA resulted in the reduction of respective TORC proteins, but only siRNA to the TORC2 gene suppressed *Gax *gene expression. Proteins were detected with antibodies to the respective TORC proteins, Tax, or β-actin by the ECL or ECL plus system. B. Densitometric analysis data of TORC proteins and Gax protein normalized to β-actin are presented as mean ± SEM of three independent experiments. T1, TORC1; T2, TORC2; T3, TORC3.

To investigate which TORC protein functioned dominantly in EL4-Gax cells, we analyzed *Gax *expression after the knock-down of the three TORC genes by transducing the cells with a retrovector for siRNA against each TORC genes. Expression of siRNA resulted in the reduction of the respective gene product by more than 50% (Fig. 5B) but a significant reduction of Gax protein expression was only observed in cells with the siRNA to the TORC2 RNA (Fig. 5A, B). We, thus, concluded that TORC2 is primarily involved in the transcriptional control of the HTLV-1 promoter in EL4-Gax cells. Together, these results suggest that the reduced TORC2 expression in EL4-Gax cells *in vivo *is closely associated with the silencing of *Gax *gene expression *in vivo*.

### Nuclear expression of TORC2 protein was reduced in EL4-Gax cells *in vivo*

**Figure 6:**
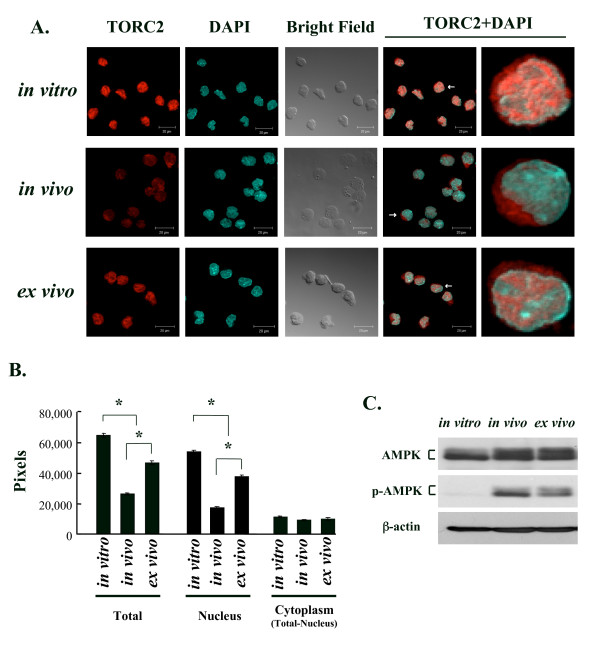
**Expression and subcellular localization of TORC2 in EL4-Gax cells**. A. Immunofluorescent staining of TORC2 protein (red) in EL4-Gax cells grown *in vitro*, *in vivo*, or *ex vivo*. Cells were counterstained with DAPI (blue) to localize the nucleus and examined by confocal microscopy. The right panel shows a magnified image of a single cell indicated by an arrow in the adjacent panel. B. Statistical analysis of subcellular localization of TORC2 expression. The amount of protein expression was determined as the number of fluorescent pixels in the total and nucleus for each growth condition. Data were obtained by counting pixels in 108, 137, and 106 cells for *in vitro*, *in vivo*, and *ex vivo *conditions, respectively. Error bars indicate SEMs. *; p < 0.001. C. Expression of AMPKα protein in EL4-Gax cells. Anti-AMPKα and anti-phosphor-AMPKα (Thr172) antibodies were used to detect protein in EL4-Gax cells grown *in vitro*, *in vivo*, and *ex vivo*. Equivalent protein loading was confirmed by stripping and re-probing the blot with an anti-β-actin antibody.

Phosphorylation of TORC2 protein by cellular kinases, such as AMPK (AMP-activated protein kinase) kinase, induces the translocation of TORC2 from the nucleus to the cytoplasm, thereby suppressing CREB-dependent transcription. In fact, the unphosphorylated form of the TORC2 protein *in vivo *appeared to be reduced more significantly than the phosphorylated form, when compared *in vitro *or *ex vivo *by Western blotting (Fig. 4B). Therefore, activity of AMPK was examined by measuring the phosphorylation at Thr172, which is required for AMPK activation [35]. The results shown in Figure 6C clearly demonstrate the activation of AMPK activity in EL4-Gax cells *in vivo *and its reduction in cells cultured *ex vivo*.

Subsequently, the subcellular localization of TORC2 in EL4-Gax cells *in vitro *and *in vivo *was examined using immunostaining (Fig. 6A). Consistent with the Western blotting, expression of the TORC2 protein in *in vivo *cells was greatly reduced in comparison with that in the *in vitro *cultured cells, and the expression was restored after *ex vivo *culture (Fig. 6A, "TORC2", and Fig. 6B). Furthermore, the subcellular localization of TORC2 was restricted to the cytoplasm of *in vivo *cells (Fig. 6A, "TORC2 + DAPI"), whereas the protein was primarily expressed in the nucleus in cells cultured *in vitro *and *ex vivo *(Fig. 6A, B).

Because cytoplasmic retention of TORC2 results in its degradation by proteasomes, it appears that some *in vivo *-specific cellular signal(s) may induce the cytoplasmic translocation, and thereby the degradation of the TORC2 protein, resulting in the suppression of HTLV-1 transcription in EL4-Gax cells.

## Discussion

Tax protein plays a key role in the development of ATL and other HTLV-1-related diseases through pleiotropic actions, that include transactivation of the NF-κB [36], CREB [22,21,24], and SRF pathways [37,38]; transrepression of lck [39], p18 [40], DNA polymerase β [41], and histone gene transcription [42]; and functional inactivation of p53 [43] and MAD1 [44]. However, the expression of viral genes, including Tax, is strongly suppressed in the peripheral blood of patients infected with HTLV-1 [12], mainly because the Tax protein harbors several strong epitopes for cytotoxic T -cells [14]. Such suppression is readily reversible, because gene expression of HTLV-1 in peripheral blood cells from infected people, with the exception of two-thirds of ATL patients [13], quickly resumes when the infected cells are moved to *in vitro *conditions, without any additional stimulation [12]. This indicates that the transient expression of Tax is essential for the propagation of viral infection, and/or the infected cells are under strict surveillance by the host immune system [15].

DNA methylation is a host defense mechanism for inactivating transposable elements, such as retroviruses, to inhibit their transcription and their generation of new viruses. Thus, the transcriptional silencing of the Tax gene has been studied extensively in terms of DNA methylation of the 5'-LTR, which is the promoter of viral transcription [45,17,13,18]. In ATL-derived cell lines, complete- or hypermethylation of CpG DNA in 5'-LTR is closely associated with the suppression of viral gene transcription, and partial methylation is not sufficient to silence transcription of the Tax gene [13,17]. In contrast, the 5'-LTR of the provirus genome was found to be unmethylated or partially methylated in about 70% of ATL leukemic cells with intact provirus sequences [18], whereas Tax gene expression was mostly silenced *in vivo*. Furthermore, lack of DNA methylation in the 5'-LTR was more frequently observed in HTLV-1 carriers than in primary ATL cells [18]. These data indicate that a mechanism other than DNA methylation is involved in the silencing of Tax gene transcription in the peripheral blood of HTLV-1 -infected individuals *in vivo*. The mouse model system studied in this report models gene control in HTLV-1 -infected cells in ATL patients and carriers, where reversible silencing of Tax gene transcription is observed, without extensive DNA methylation of the 5'-LTR.

The transcription of HTLV-1 is controlled by CREB binding to the enhancer sequence, which consists of three 21-bp direct repeats containing CREs in the middle. The viral transactivator, Tax, interacts with both CREB and CBP/p300 in a manner independent of the phosphorylation of CREB and activates the HTLV-1 enhancer. In the current study, approximately equivalent binding of CREB and CBP on the enhancer DNA was observed in the *Gax *expressing EL4-Gax cells cultured *in vitro *or EL4-Gax cells grown *in vivo*, where *Gax *expression was substantially suppressed (Fig. 3B and Fig. 1D). In addition, although histone acetylation, a downstream effect of CREB-CBP/p300 association, remained constant, recruitment of the basic transcription factor (TFIIB) and RNA polymerase II to the promoter was diminished in cells grown *in vivo *(Fig. 3C). These observations suggest that activation machinery other than the CREB-CBP/p300 pathway might be involved in the transcriptional control of HTLV-1 *in vivo*, whereby constitutive association of CREB-CBP/p300 and acetylated histones keeps the promoter in an inducible condition. In this respect, it is noteworthy that reversible silencing was observed in an ATL case regardless of hyperacetylation of histones H3 and H4 in the 5'-LTR [18].

CREB-dependent promoters have been thought to respond to various intracellular and extracellular signals via stimulus-dependent phosphorylation of CREB at Ser-133 and the resulting recruitment of the co-activator paralogues, CBP and p300 [46]. Although a number of growth factors and hormones trigger CREB phosphorylation with comparable stoichiometry and kinetics, they are often ineffective in promoting transcription through the CREB binding site. Furthermore, knock-in mice with mutations in CBP/p300 that blocked the interaction with phosphor-CREB showed only modest changes in cAMP-dependent transcription. Thus, the involvement of additional CREB co-activators was postulated, and, subsequently, a new family of proteins has been identified as co-activators independent of CREB phosphorylation using high-throughput transformation assays [25,26]. The novel co-activators, TORC 1–3, interact with the bZIP of CREB in a phospho-Ser133-independent manner through their N-terminal coiled-coil structure, leading to the activation of CREB -mediated transcription.

Accordingly, we examined the involvement of TORCs in the regulation of HTLV-1 transcription *in vivo *and found that expression of TORC1 and TORC2 in EL4-Gax cells coincided with the suppressed expression of *Gax in vivo *and its up-regulation *in vitro *or *ex vivo*. Previous reports have demonstrated that the forced expression of human TORC proteins activates the transcription of the HTLV-1 LTR, whereas depletion of TORCs by siRNA-mediated knock-down impairs Tax-dependent and Tax-independent transcription [27,28]. We also observed the activation of a luciferase reporter gene under control of HTLV-1 LTR in association with the over-expression of TORC1, TORC2, or TORC3 of mouse origin (data not shown). It was recently reported that TORC3 repressed the transcription of HTLV-1 by interacting with Bcl3, which, in turn, recruits HDAC1 to deacetylate histones. However, such a mechanism is likely not involved in the current system because no significant change in the promoter association of acetylated histones H3 and H4 was observed in cells grown *in vitro *or *in vivo*, although the expression of TORC3 appeared to increase slightly *in vivo*. In contrast, depletion of TORC1 or TORC3 expression in EL4-Gax cells by siRNA did not affect the expression of *Gax*, whereas TORC2 knock-down resulted in a significant reduction in *Gax *expression, although expression of siRNA to any of the TORC genes reduced the amount of the respective TORC proteins in EL4-Gax cells to about the same extent. Consistently, the analysis of enhancer binding of TORC proteins by using a ChIP assay revealed that TORC2 is the major binding factor among the three TORC-family proteins in EL4-Gax cells (Fig. 4E). These results indicate that TORC2 is primarily responsible for the transcriptional control of HTLV-1 LTR in EL4-Gax cells *in vivo*.

TORC2 activity is regulated by phosphorylation of the protein, which induces the cytoplasmic translocation of TORC2 from the nucleus, leading to its degradation by the 26S proteasome. In fact, we demonstrated that the activity of AMPK, a family of Ser/Thr kinases, which phosphorylates TORC2 [29,35], was activated in EL4-Gax cells *in vivo *(Fig. 6C). In EL4-Gax cells cultured *in vitro*, most TORC2 protein was localized in the nucleus where it would be involved in the transcription of the provirus. When the cells were grown in the peritoneal cavity of mice, the nuclear expression of TORC2 was markedly diminished, whereas its cytoplasmic expression remained relatively constant. This result is consistent with the Western blotting data, which showed that the amount of the unphosphorylated form of TORC2 protein was more significantly reduced *in vivo *than that of the phosphorylated form (Fig. 4B). Together, these findings suggest that some signal(s) specific for *in vivo *growth induces the phosphorylation of TORC2 protein, leading to the cytoplasmic translocation and degradation of the protein. In this respect, it is noteworthy that the activity of AMPK, is modulated by various pathological stresses and physiological stimuli, including glucose deprivation, hyperosmotic stress, heat shock, hypoxia, and ischemia [35]. In addition, calcium influx after hormonal stimulation or antigen stimulation of lymphocytes induces calcineurin activity, which in turn dephosphorylates TORC2. Thus, various physiological and immunological signals can induce the reactivation of provirus, even *in vivo*, through modulation of TORC2 activity.

## Conclusion

Using a mouse model system in which the transcriptional control was quite similar to that in asymptomatic HTLV-1 -infected carriers [30], we demonstrated that TORC2, a recently identified co-activator of CREB, is a key determinant of HTLV-1 gene expression *in vivo*. An analysis of promoter binding by transcriptional factors and chromatin modifications in EL4-Gax cells revealed that neither CpG methylation of the HTLV-1 LTR nor reduced association of enhancer binding factors such as the CREB-CBP/p300 complex coincided with the *in vivo *-specific suppression of HTLV-1 transcription. Instead, the expression of TORC2 was coordinated with suppression *in vivo *and the reactivation *ex vivo *of provirus transcription. In addition, the transcriptional activities of the provirus gene *in vivo *and *in vitro *were associated with the nuclear accumulation of TORC2 protein, which is tightly controlled by phosphorylation of the protein in response to various physiological signals.

## Methods

### Cell lines and animal model

The establishment of the EL4-Gax mouse model system was previously reported [30]. Briefly, 1 × 10^6 ^EL4-Gax cells were injected into the peritoneal cavity of 6-week-old male C57BL/6J mice (Charles River Laboratories Japan, Kanagawa, Japan) in 0.1 mL phosphate -buffered saline (PBS). After three weeks, cells in ascitic fluids (*in vivo *cells) were recovered and cultured in culture medium for 48 h (*ex vivo *culture). *Gax *expression was monitored using a FACS (Becton Dickinson, NJ) as the intensity of GFP fluorescence. The mice were maintained under standard pathogen-free conditions in the animal facility of Kansai Medical University, and the protocol for the experiments was approved by the Institutional Animal Care and Use Committee of Kansai Medical University.

### Quantitative real-time RT-PCR analysis of *Gax *gene transcripts

Total RNA was extracted from cells using the Trizol reagent (Invitrogen, Carlsbad, CA) and treated with RNase-free DNase I (GIBCO BRL) at 37°C for 30 min, followed by phenol-chloroform extraction and ethanol precipitation. Total RNA (1 μg) was subjected to reverse transcription using RivaTra Ace (Toyobo, Osaka, Japan) with random 9 mer oligonucleotides (Takara, Kyoto, Japan) as a primer.

Real-time PCR was performed in a 25-μL reaction mixture consisting of 2-fold SYBR Green mastermix (Applied Biosystems, UK); 50 pg or 10 ng cDNA for 18S-rRNA or *Gax*, respectively; 0.4 μM primer; and the BioRad-iQ-Real-Time PCR Detection System. The cycling conditions for all amplifications were 95°C for 15 min; 40 cycles of 95°C for 30 s, 60°C for 30 s, and 72°C for 30 s, with a single fluorescence measurement melting curve program (60–95°C with a heating rate of 0.2°C per second and a continuous fluorescence measurement); and finally a cooling step to 24°C. Each individual sample was run in triplicate wells; the cycle threshold (Ct) of each well was recorded at the end of the reaction. The Ct was manually set up at the level that reflected the best kinetic PCR parameters, and melting curves were analyzed. The average and standard deviation (SD) of the three Cts was calculated, and the average value was accepted if the SD was less than 0.38. The 2^ΔΔ^Ct method was used to analyze the relative changes in *Gax *gene expression in *in vivo *or *ex vivo *samples with those *in vitro*.

The primers used for PCR were as follows: for the *Gax *gene, 5'-ACGCCTATGATTTCCGGGCC-3' and 5'-GGATATTTGGGGCTCATGGTCA-3'; for CD4, 5'-CAGAGCCTGACCCTGACCTTG-3' and 5'-CATCACCACCAGGTTCACTTCC-3'; for endogenouse mouse retroviral env, 5'-GAAGGTCCAGCGTTCTCAAAAT-3' and 5'-CACGTGATTTCACTTCTTCTGG-3'[47]; for elongation factor (EF)-1α, 5'-TCTGGTTGGAATGGTGACAACATGC-3' and 5'-CCAGGAAGAGCTTCACTCAAAGCTT-3'; for GAPDH, 5'-ACCACAGTCCATGCCATCAC-3' and 5'-TCCACCACCCTGTTGCTGTA-3'; for β-actin: 5'-GAGATCTGCCGATCCGCCGCCCG-3', and 5'-GCTCGAGGTGTGCACTTTTATTCAACTGG-3'; for 18S-rRNA, 5'-CTCGATTCCGTGGGTGGTGGTG-3' and 5'-GGCCGATCCGAGGGCCTCAC-3'.

### Western blot analysis

Cells were lysed and sonicated in cell lysis buffer (20 mM Tris-HCl [pH 7.5], 150 mM NaCl, 1 mM Na_2_EDTA, 1 mM EGTA, 1%Triton X-100, Cell Signaling Technology, MA) plus complete proteinase inhibitor cocktail and phosphatase inhibitor mixture (Roche Applied Science, Indianapolis, IN). Aliquots containing 5–30 μg of protein were separated by SDS/polyacrylamide gel electrophoresis; transferred onto PVDF membrane (Immobilon-P, Millipore, Billerica, MA); and detected by the ECL or ECL plus system (GE Healthcare, Buckinghamshire, UK). Used antibodies were TORC2 rabbit polyclonal antibody (#ST1099, Calbiochem, La Jolla, CA), TORC1 rabbit polyclonal antibody (#2501), TORC3 rabbit polyclonal antibody (#2768), phospho-AMPKα (Thr172) rabbit monoclonal antibody (40H9, #2535), AMPKα rabbit monoclonal antibody (23A3, #2603, Cell Signaling Technology); CREB1 (#06–863), phospho-CREB (#06–519, Upstate Technology, NY); PARP-1 mouse monoclonal antibody (#611038, BD. Biosciences, San Jose, CA) or β-actin mouse monoclonal antibody (clone AC-5, Sigma-Aldrich, MO) and HRP-conjugated protein A for rabbit primary antibodies or anti-mouse IgG-HRP (GE Healthcare) for mouse monoclonal primary antibodies.

### Phosphatase treatment of protein from EL4-Gax

1 × 10^6 ^EL4-Gax cells were lysed in 250 μl of cell lysis buffer (mentioned above, Cell Signaling Technology) and 1× complete protease inhibitor cocktail (Roche) with or without phosphatase inhibitor cocktail (2.5 mM sodium pyrophosphate; 1 mM β-glycerophosphate; 1 mM Na_3_VO_4_; 50 mM sodium fluoride), plugged into liquid nitrogen and thawed on ice immediately. Lysate was clarified by centrifugation at 14,000 rpm for 10 minutes at 4°C. After measuring protein concentration, 20 μg protein was dispended to 1× rAPid Alkaline Phosphatase buffer with or without phosphatase inibitor cocktail, treated with or without 20 units of rAPid Alkaline Phosphatase (Cat No.04898133001, Roche) for 60 minutes at 37°C in a 100 μl reaction volumes. The 1/3 volume of samples was then separated by SDS-PAGE.

### Analysis of CpG methylation

Methylation of the cytosine residue at CpG sites was analyzed using the bisulfite genomic sequencing method [16] with minor modifications. Briefly, 1 μg genomic DNA was used for bisulfite treatment. The DNA sample in 0.2 N NaOH was denatured at 37°C for 10 min, followed by incubation at 50°C overnight in 2.6 M sodium bisulfite and 0.5 mM hydroquinone (Sigma-Aldrich) solution. The solution was then sealed with 200 μL mineral oil and kept in the dark. Sample DNA was purified using the Wizard DNA Clean-Up system (Promega, Madison, WI) and treated with 0.3 N NaOH. DNA was precipitated with ethanol and dissolved in 50 μL H_2_O. Then 30% of this solution was subjected to PCR.

PCR was performed in 50-μL reaction mixtures containing 1 μL Acuprime Taq DNA polymerase and its buffer (Invitrogen) and a primer set at 0.2 μM. The primer sequences to amplify the 5'-LTR HTLV-1 U3 region were sense primer (complementary to the modified cellular flanking region), 5'-CCTCCTATCAAAACTAACTTTAAC-3', and antisense primer (complementary to the modified HTLV-1 U3 region), 5'-AAGTTTTTTGAGGTGAGGGGT-3'.

The cycling conditions for all amplifications were 94°C for 2 min; 40 cycles of 94°C for 30 s, 56°C for 30 s, and 68°C for 30 s; and a final extension at 68°C for 5 min. The amplified PCR products, purified with the QIA quick PCR kit (QIAGEN, Germantown, MD), were subcloned into a pGEM-T easy vector (Promega), and the nucleotide sequences of at least 13 clones were determined.

### ChIP assay

ChIP assays were performed according to the manufacturer's protocol (Upstate Biotechnology, Lake Placid, NY) with some modifications. Briefly, 2 × 10^6 ^cells were fixed with 1% formaldehyde for 5 min at room temperature and sonicated (Biorupter UCD-200T; CosmoBio, Tokyo, Japan) to obtain on average 600 -bp length of soluble chromatin. The chromatin solutions were pre-cleared with 80 μL 50% Protein G Sepharose slurry (Amersham) preabsorbed with 0.2 mg/mL sonicated salmon sperm DNA and 0.05% BSA. After a 10-fold dilution with ChIP dilution buffer (0.01% SDS, 1.1% Triton X-100, 1.2 mM EDTA, 16.7 mM Tris-HCl, pH 8.1, 167 mM NaCl), 2 mL pre-cleared complex was combined with 2–15 μg antibodies against CREB1 (06–863), phosphor-CREB (06–519), acetylated H3 at Lys-9, 14 (06–599), acetylated H4 at Lys-5, 8, 12, 16 (06–598, Upstate Technology), CBP (sc369), TFIIB (sc-225), Pol-II (sc899, Santa Cruz Biotechnology, CA), methylated H3 Lys-4 (AB8580, Abcam Ltd., Cambridge, UK), GFP (mouse monoclonal antibody, clones 7.1 and 13.1, Boehringer Mannheim), or normal mouse IgG (I-5381, Sigma), rabbit IgG (011-000-002, Jackson immunoresearch Laboratories, West Grove, PA) and rotated at 4°C overnight. The immune complexes were collected by incubating with 80 μL of protein G slurry. DNA-protein cross-linking was reversed by incubation at 65°C overnight, and the samples were treated with RNase A and then with proteinase K. After phenol extraction and ethanol precipitation, the DNA was finally dissolved into 30 μL of water.

The primers used for PCR were as follows. For the 5'-LTR enhancer, sense primer, complementary to the 5'flanking cellular region, 5'-AAGCACAGAAGACACCTTGTGCAC-3' and antisense primer, complementary to 5'-LTR HTLV-1 U3 region, 5'-AAGTTTTTTGAGGTGAGGGGTTGTCG-3'. 5'-LTR promoter region, sense primer, complementary to HTLV-1 U3/MLV U3 junction region, 5'-CATGGCACGCATATGTCTAGAGAACC-3', antisense primer, complementary to MLV gag region, 5'-TCTCCCGATCCCGGACGAGCC-3'. The following sequences of primers were used for other control gene promoters: for EF1a, f (-20): 5'-CGAGGGTGGGGGAGAACGGTAT-3' and r (152): 5'-AGCTAATCCCCGCCGACGACAG-3'; for β-globin, f (-117): 5'-ACCGAAGCCTGATTCCGTAGAGC-3' and r (+133): 5'-CTCACCACCAACTTCATCGGAGTT-3'.

All PCRs were performed in 20-μL reaction mixes consisting of 1–10 μL of the ChIP product, 1 U Ex Taq polymerase (Takara), 200 mM each of deoxynucleoside triphosphates, and a primer set at 0.4 μM. The cycling conditions for all amplifications were 94°C for 5 min; 33–35 cycles of 94°C for 30 s, 62–68°C for 30 s, and 72°C for 30 s; and a final extension at 72°C for 5 min.

### Immunofluorescent staining

Cells were grown in suspension or harvested from ascites and were then fixed by adding an equal volume of 4% paraformaldehyde diluted in PEM/PBS(-) buffer (1/5 volume of PEM [see below] and 4/5 volume of PBS(-)) for 30 min at 4°C, collected by centrifugation (300*g*, 3 min), washed and resuspended in PBS(-), and then cytospun (Shandon Cytospin3; 20,000 cells/slide, 700 rpm, 1 min) onto 1 N HCl and 95% ethanol-washed slide glasses (Matsunami, Osaka, Japan). The cells were washed twice with PEM buffer (80 mM potassium PIPES [piperazine-*N*, *N*'-bis(2-ethanesulfonic acid)] [pH 6.8], 5 mM EGTA [pH 7.0], 2 mM MgCl2) and permeabilized by incubation in PEM/PBS(-) buffer containing 0.1% Triton X-100 for 30 min at room temperature and blocked with goat serum (1:20 dilution in TBS containing 0.1% Tween-20) for 30 min at room temperature. After blocking, the cells were incubated with anti-TORC2 antiserum ((#ST1099, Calbiochem) or normal rabbit IgG for 3 h at room temperature or overnight at 4°C in TBS containing 0.1% Tween-20 (TBS-T). Thereafter, the cells were washed three times with TBS-T and incubated with Cy5-conjugated AffiniPure donkey anti-rabbit IgG (#711-175-152, Jackson Immuno Research Laboratories) in TBS-T. After 1 h, the cells were washed three times in TBS-T. To visualize the nucleus, we counterstained the cells with DAPI (4', 6'-diamidino-2-phenylindole; Nacalai Tesque, Kyoto, Japan; final concentration 0.4 μg/mL, 5 min incubation at room temperature) and washed them once with PBS(-), then mounted them in mounting media and examined them with a Zeiss LSM510 META microscope.

### siRNA interference

TORC siRNA hairpin expression vectors (shTORC1 retroviral vector, #NM-001004062; shTORC3 retroviral vector, #XM-344915; shTORC2 lentiviral vector, #NM-02881) were purchased from Open Biosystems (Huntsville, AL). Recombinant retroviruses for shTORC1 and shTORC3 were generated by transfecting respective retroviral vector plasmids into BOSC23 cells, and recombinant lentivirus expressing shTORC2 was generated in HEK293T cells by the cotransfection of the lentiviral vector plasmid with expression vectors for Gag/Pol (pGag/Pol), Rev (pRev), and VSV-G protein (pVSV-G) using the Lipofectamine 2000 (Invitrogen) transfection reagent. Viral supernatant was collected 48 h after transfection and added to EL4-Gax cells for infection in the presence of polybrene (8 μg/mL). Infected cells were selected with puromycin (4 μg/mL), and mixtures of 40–200 clones of each virus were used for further analysis.

## Competing interests

The authors declare that they have no competing interests.

## Authors' contributions

SJ designed and carried out most of the experiments. TI conceived the mouse model system and performed the FACS analysis. MT assisted with the experiment in Figure 5 and analyzed the data. FRA established the EL4-Gax cells and participated in the RT-PCR and DNA methylation assay. KS participated in the establishment of the mouse model system and data analysis. JF directed and supervised the experiments and interpretation. All authors have read and approved the final manuscript.

## References

[B1] Yoshida M, Miyoshi I, Hinuma Y (1982). Isolation and characterization of retrovirus from cell lines of human adult T-cell leukemia and its implication in the disease. Proc Natl Acad Sci U S A.

[B2] Poiesz BJ, Ruscetti FW, Gazdar AF, Bunn PA, Minna JD, Gallo RC (1980). Detection and isolation of type C retrovirus particles from fresh and cultured lymphocytes of a patient with cutaneous T-cell lymphoma. Proc Natl Acad Sci USA.

[B3] Osame M, Usuku K, Izumo S, Ijichi N, Amitani H, Igata A, Matsumoto M, Tara M (1986). HTLV-I associated myelopathy, a new clinical entity. Lancet.

[B4] Gessain A, Barin F, Vernant JC, Gout O, Maurs L, Calender A, de The G (1985). Antibodies to human T-lymphotropic virus type-I in patients with tropical spastic paraparesis. Lancet.

[B5] Seiki M, Hattori S, Hirayama Y, Yoshida M (1983). Human adult T-cell leukemia virus: complete nucleotide sequence of the provirus genome integrated in leukemia cell DNA. Proc Natl Acad Sci USA.

[B6] Sodroski JG, Rosen CA, Haseltine WA (1984). Trans-acting transcriptional activation of the long terminal repeat of human T lymphotropic viruses in infected cells. Science.

[B7] Cann AJ, Rosenblatt JD, Wachsman W, Shah NP, Chen IS (1985). Identification of the gene responsible for human T-cell leukaemia virus transcriptional regulation. Nature.

[B8] Felber BK, Paskalis H, Kleinman-Ewing C, Wong-Staal F, Pavlakis GN (1985). The pX protein of HTLV-I is a transcriptional activator of its long terminal repeats. Science.

[B9] Fujisawa J, Seiki M, Kiyokawa T, Yoshida M (1985). Functional activation of the long terminal repeat of human T-cell leukemia virus type I by a trans-acting factor. Proc Natl Acad Sci USA.

[B10] Yoshida M (2001). Multiple viral strategies of HTLV-1 for dysregulation of cell growth control. Annu Rev Immunol.

[B11] Boxus M TJ, Legros S, Dewulf JF, Kettmann R, Willems L (2008). The HTLV-1 Tax interactome. Retrovirology.

[B12] Tochikura T, Iwahashi M, Matsumoto T, Koyanagi Y, Hinuma Y, Yamamoto N (1985). Effect of human serum anti-HTLV antibodies on viral antigen induction in vitro cultured peripheral lymphocytes from adult T-cell leukemia patients and healthy virus carriers. Int J Cancer.

[B13] Takeda S, Maeda M, Morikawa S, Taniguchi Y, Yasunaga J, Nosaka K, Tanaka Y, Matsuoka M (2004). Genetic and epigenetic inactivation of tax gene in adult T-cell leukemia cells. Int J Cancer.

[B14] Kannagi M, Harada S, Maruyama I, Inoko H, Igarashi H, Kuwashima G, Sato S, Morita M, Kidokoro M, Sugimoto M (1991). Predominant recognition of human T cell leukemia virus type I (HTLV-I) pX gene products by human CD8+ cytotoxic T cells directed against HTLV-I-infected cells. Int Immunol.

[B15] Kannagi M, Harashima N, Kurihara K, Ohashi T, Utsunomiya A, Tanosaki R, Masuda M, Tomonaga M, Okamura J (2005). Tumor immunity against adult T-cell leukemia. Cancer Sci.

[B16] Bangham CR (2003). Human T-lymphotropic virus type 1 (HTLV-1): persistence and immune control. Int J Hematol.

[B17] Koiwa T, Hamano-Usami A, Ishida T, Okayama A, Yamaguchi K, Kamihira S, Watanabe T (2002). 5'-long terminal repeat-selective CpG methylation of latent human T-cell leukemia virus type 1 provirus in vitro and in vivo. J Virol.

[B18] Taniguchi Y, Nosaka K, Yasunaga J, Maeda M, Mueller N, Okayama A, Matsuoka M (2005). Silencing of human T-cell leukemia virus type I gene transcription by epigenetic mechanisms. Retrovirology.

[B19] Fujisawa J, Seiki M, Sato M, Yoshida M (1986). A transcriptional enhancer sequence of HTLV-I is responsible for trans-activation mediated by p40 chi HTLV-I. Embo J.

[B20] Yoshimura T, Fujisawa J, Yoshida M (1990). Multiple cDNA clones encoding nuclear proteins that bind to the tax-dependent enhancer of HTLV-1: all contain a leucine zipper structure and basic amino acid domain. Embo J.

[B21] Suzuki T, Fujisawa JI, Toita M, Yoshida M (1993). The trans-activator tax of human T-cell leukemia virus type 1 (HTLV-1) interacts with cAMP-responsive element (CRE) binding and CRE modulator proteins that bind to the 21-base-pair enhancer of HTLV-1. Proc Natl Acad Sci USA.

[B22] Zhao LJ, Giam CZ (1992). Human T-cell lymphotropic virus type I (HTLV-I) transcriptional activator, Tax, enhances CREB binding to HTLV-I 21-base-pair repeats by protein-protein interaction. Proc Natl Acad Sci USA.

[B23] Lenzmeier BA, Giebler HA, Nyborg JK (1998). Human T-cell leukemia virus type 1 Tax requires direct access to DNA for recruitment of CREB binding protein to the viral promoter. Mol Cell Biol.

[B24] Kwok RP, Laurance ME, Lundblad JR, Goldman PS, Shih H, Connor LM, Marriott SJ, Goodman RH (1996). Control of cAMP-regulated enhancers by the viral transactivator Tax through CREB and the co-activator CBP. Nature.

[B25] Iourgenko V, Zhang W, Mickanin C, Daly I, Jiang C, Hexham JM, Orth AP, Miraglia L, Meltzer J, Garza D (2003). Identification of a family of cAMP response element-binding protein coactivators by genome-scale functional analysis in mammalian cells. Proc Natl Acad Sci USA.

[B26] Conkright MD, Canettieri G, Screaton R, Guzman E, Miraglia L, Hogenesch JB, Montminy M (2003). TORCs: transducers of regulated CREB activity. Mol Cell.

[B27] Siu YT, Chin KT, Siu KL, Yee Wai Choy E, Jeang KT, Jin DY (2006). TORC1 and TORC2 coactivators are required for tax activation of the human T-cell leukemia virus type 1 long terminal repeats. J Virol.

[B28] Koga H, Ohshima T, Shimotohno K (2004). Enhanced activation of tax-dependent transcription of human T-cell leukemia virus type I (HTLV-I) long terminal repeat by TORC3. J Biol Chem.

[B29] Screaton RA, Conkright MD, Katoh Y, Best JL, Canettieri G, Jeffries S, Guzman E, Niessen S, Yates JR, Takemori H (2004). The CREB coactivator TORC2 functions as a calcium- and cAMP-sensitive coincidence detector. Cell.

[B30] Furuta RA, Sugiura K, Kawakita S, Inada T, Ikehara S, Matsuda T, Fujisawa J (2002). Mouse model for the equilibration interaction between the host immune system and human T-cell leukemia virus type 1 gene expression. J Virol.

[B31] Kaufmann SH, Desnoyers S, Ottaviano Y, Davidson NE, Poirier GG (1993). Specific proteolytic cleavage of poly(ADP-ribose) polymerase: an early marker of chemotherapy-induced apoptosis. Cancer Res.

[B32] Lazebnik YA, Kaufmann SH, Desnoyers S, Poirier GG, Earnshaw WC (1994). Cleavage of poly(ADP-ribose) polymerase by a proteinase with properties like ICE. Nature.

[B33] Kim YM, Ramirez JA, Mick JE, Giebler HA, Yan JP, Nyborg JK (2007). Molecular characterization of the Tax-containing HTLV-1 enhancer complex reveals a prominent role for CREB phosphorylation in Tax transactivation. J Biol Chem.

[B34] Chi T, Lieberman P, Ellwood K, Carey M (1995). A general mechanism for transcriptional synergy by eukaryotic activators. Nature.

[B35] Hawley SA, Davison M, Woods A, Davies SP, Beri RK, Carling D, Hardie DG (1996). Characterization of the AMP-activated protein kinase kinase from rat liver and identification of threonine 172 as the major site at which it phosphorylates AMP-activated protein kinase. J Biol Chem.

[B36] Leung K, Nabel GJ (1988). HTLV-1 transactivator induces interleukin-2 receptor expression through an NF-kappa B-like factor. Nature.

[B37] Suzuki T HH, Fujisawa J, Fujita T, Yoshida M (1993). A trans-activator Tax of human T-cell leukemia virus type 1 binds to NF-kappa B p50 and serum response factor (SRF) and associates with enhancer DNAs of the NF-kappa B site and CArG box. Oncogene.

[B38] Fujii M TH, Chuhjo T, Minamino T, Miyamoto K, Seiki M (1994). Serum response factor has functional roles both in indirect binding to the CArG box and in the transcriptional activation function of human T-cell leukemia virus type I Tax. J Virol.

[B39] Lemasson I R-HV, Hamaia S, Duc Dodon M, Gazzolo L, Devaux C (1997). Transrepression of lck gene expression by human T-cell leukemia virus type 1-encoded p40tax. J Virol.

[B40] Suzuki T, Narita T, Uchida-Toita M, Yoshida M (1999). Down-regulation of the INK4 family of cyclin-dependent kinase inhibitors by tax protein of HTLV-1 through two distinct mechanisms. Virology.

[B41] Jeang KT WS, Semmes OJ, Wilson SH (1990). HTLV-I trans-activator protein, tax, is a trans-repressor of the human beta-polymerase gene. Science.

[B42] Bogenberger JM, Laybourn PJ (2008). Human T Lymphotropic Virus Type 1 protein Tax reduces histone levels. Retrovirology.

[B43] Uittenbogaard MN GH, Reisman D, Nyborg JK (1995). Transcriptional repression of p53 by human T-cell leukemia virus type I Tax protein. J Biol Chem.

[B44] Jin  DY, Spencer F, Jeang KT (1998). Human T cell leukemia virus type 1 oncoprotein Tax targets the human mitotic checkpoint protein MAD1. Cell.

[B45] Saggioro  D, Panozzo PM, Chieco-Bianchi L (1990). Human T-lymphotropic virus type I transcriptional regulation by methylation. Cancer Res.

[B46] Chrivia JC, Kwok RP, Lamb N, Hagiwara M, Montminy MR, Goodman RH (1993). Phosphorylated CREB binds specifically to the nuclear protein CBP. Nature.

[B47] Cho BC SJJ, Largaespada DA, Bedigian HG, Buchberg AM, Jenkins NA, Copeland NG (1995). Frequent disruption of the Nf1 gene by a novel murine AIDS virus-related provirus in BXH-2 murine myeloid lymphomas. J Virol.

